# Caspase Dependent Programmed Cell Death in Developing Embryos: A Potential Target for Therapeutic Intervention against Pathogenic Nematodes

**DOI:** 10.1371/journal.pntd.0001306

**Published:** 2011-09-13

**Authors:** Alok Das Mohapatra, Sunil Kumar, Ashok Kumar Satapathy, Balachandran Ravindran

**Affiliations:** 1 Department of Infectious Disease Biology, Institute of Life Sciences, DBT, Ministry of Science and Technology, Government of India, Bhubaneswar, India; 2 Department of Applied Immunology, Regional Medical Research Centre, Indian Council of Medical Research, Government of India, Bhubaneswar, India; McGill University, Canada

## Abstract

**Background:**

Successful embryogenesis is a critical rate limiting step for the survival and transmission of parasitic worms as well as pathology mediated by them. Hence, blockage of this important process through therapeutic induction of apoptosis in their embryonic stages offers promise for developing effective anti-parasitic measures against these extra cellular parasites. However, unlike in the case of protozoan parasites, induction of apoptosis as a therapeutic approach is yet to be explored against metazoan helminth parasites.

**Methodology/Principal Findings:**

For the first time, here we developed and evaluated flow cytometry based assays to assess several conserved features of apoptosis in developing embryos of a pathogenic filarial nematode *Setaria digitata*, in-vitro as well as ex-vivo. We validated programmed cell death in developing embryos by using immuno-fluorescence microscopy and scoring expression profile of nematode specific proteins related to apoptosis [e.g. CED-3, CED-4 and CED-9]. Mechanistically, apoptotic death of embryonic stages was found to be a caspase dependent phenomenon mediated primarily through induction of intracellular ROS. The apoptogenicity of some pharmacological compounds viz. DEC, Chloroquine, Primaquine and Curcumin were also evaluated. Curcumin was found to be the most effective pharmacological agent followed by Primaquine while Chloroquine displayed minimal effect and DEC had no demonstrable effect. Further, demonstration of induction of apoptosis in embryonic stages by lipid peroxidation products [molecules commonly associated with inflammatory responses in filarial disease] and demonstration of in-situ apoptosis of developing embryos in adult parasites in a natural bovine model of filariasis have offered a framework to understand anti-fecundity host immunity operational against parasitic helminths.

**Conclusions/Significance:**

Our observations have revealed for the first time, that induction of apoptosis in developing embryos can be a potential approach for therapeutic intervention against pathogenic nematodes and flow cytometry can be used to address different issues of biological importance during embryogenesis of parasitic worms.

## Author Summary

Pathogenic nematodes currently infect billions of people around the world and pose serious challenges to the economic welfare and public health in most developing countries. At present, limitations of existing therapies warrant identification of new anti-parasitic drugs/drug targets to effectively treat and control neglected tropical diseases [NTD] caused by nematode pathogens. The current gold standard for measuring/screening drug effectiveness against most helminth parasites is in-vitro assessment of motility of parasites/larvae and larval development assays which fails to provide any conclusive idea about the precise mechanism of death of parasitic worms or their larval stages. Given the huge load of parasites or their larval stages in an infected host, a compound which shows promise in in-vitro/motility screening assays but induces necrotic death in parasites/larvae will be of limited use, as it may elicit severe inflammatory response in infected hosts. In this context, the present study, which demonstrates induction of apoptotic death in developing embryos of a pathogenic nematode as a potential drug target for the first time, and provides scope for high throughput screening of pharmacological agents for their apoptogenicity against nematode embryos, is a step forward to develop novel anti-parasitic measures to challenge NTD caused by nematode pathogens.

## Introduction

Helminth infections account for the highest burden of neglected tropical diseases [NTD], which afflict the most impoverished population of the world. About two billion people are presently infected with these parasites while many more people living in endemic areas are at risk of acquiring these infections [1]-[3]. Chronic diseases caused by such metazoan parasites often inflict crippling morbidity and debilitating disability with profound economic, social and political consequences [1]. Accumulating evidence in literature suggests that coinfection with these helminth parasites increases susceptibility to or worsens progression of three major infectious diseases- HIV/AIDS, tuberculosis and malaria [4], [5]. The above facts accentuate the need for launching a global assault on parasitic worms. However, our ability to control diseases caused by these class of parasites is constrained by several factors including a limited repertoire of sub-optimal drugs and paucity of robust tools to investigate biology of nematode parasites [6].

The burden of human helminthiasis is mostly attributed to high prevalence diseases caused by pathogenic nematodes i.e. by intestinal nematodes - Ascariasis [807 million], Trichuriasis [604 million], Hook worm infections [574 million] and filarial nematodes - Lymphatic filariasis [120 million] and Onchocerciasis [37 million] respectively[1], [7]. Preventive chemotherapy through MDA programs is the mainstay for treatment and control of diseases caused by nematode pathogens, at present. However, constraints of currently available therapies including high cost, low therapeutic efficacy, rapid reinfection after treatment, poor safety profiles and patient compliance and emerging or existing drug resistance coupled with lack of robust biomarkers for detection of resistance of nematode parasites to the mainstay drugs of MDA programs etc. limit the utility of existing drugs [4], [8]–[12]. The problem is further compounded by the fact that none of the existing drugs are effective against all the life stages of the parasitic worms and almost all of them are remarkably ineffective against adult stage parasites [6], [8]. Additionally, poor understanding of the mode of action, pharmacology [6], [8], [13] and adverse side reactions associated with the front line antifilarial drugs e.g. Diethylcarbamazine **[DEC]** and Ivermectin [13], [14] etc. are issues of great concern. On the other hand, a number of studies on human immune responses to helminthic infections have been carried out so far, but no clear immune effector mechanism has emerged on which an effective vaccine could be designed against these parasites [2], [15]. Hence, control of these major tropical diseases warrants radically new approaches to chemotherapy as well as vaccine design.

Induction of apoptosis in parasites for drug development is a novel possibility that has been explored in great detail in several unicellular pathogens [16] but has not been evaluated so far, against metazoan helminth parasites. As a form of programmed cell death, apoptosis was initially described and extensively studied in the free living soil nematode *C.elegans.* Homologs of mammalian apoptosis related proteins have been identified in a trematode parasitic worm Schistosoma and function of some of these proteins has been studied by over-expressing them in artificial systems viz., mammalian cells [17]. However, no attempts have been made so far, to demonstrate functional apoptosis in parasitic worms. Presence of a thin and transparent cuticle, availability of complete genetic database with detailed molecular analysis and standardized protocols for prolonged maintenance in artificial culture medium [without requirement of susceptible animal hosts, as in case of pathogenic nematodes], have made dissection of pathways of cell death possible in free living nematode *C.elegans*. Pathogenic nematodes however, don't offer such a luxury to study apoptosis in them. Unlike free living nematodes, pathogenic nematodes of vertebrates have evolved distinct life cycles and engrossed numerous adaptations [e.g. modification of cuticle, elaborate reproductive capacity with enormous fecundity, optimum temperature for growth matching their vertebrate hosts i.e. close to 37°C as opposed to 15–25°C for *C.elegans* etc.] to suit their parasitic mode of life. Further, pathogenic nematodes very often establish chronic infections and are exposed to a volley of stress inducing factors/molecules inside their vertebrate hosts which can potentially trigger distinct pathway of apoptosis in them. Thus, induction of apoptosis in metazoan parasites might be an important arm of host immunity operational in helminth infections which is yet to be established in literature. Successful embryogenesis is a critical rate limiting step for the survival and transmission of parasitic worms as well as pathology mediated by them [18]. Hence, blockage of this important process through therapeutic induction of apoptosis in their developing embryos offers a promising approach for developing intervention strategies against these extra cellular parasites and underscores the need for studying apoptotic pathways in pathogenic nematodes. There have been no attempts so far, to study programmed cell death in nematode parasites, primarily due to lack of sensitive assay systems and the present study attempts to fill this lacuna.

We have used here *Setaria digitata*, a bovine filarial parasite as a model to demonstrate induction of apoptosis in developing embryos i.e. eggs and larvae [L-1] of a pathogenic nematode, using confocal microscopy and flow cytometry based quantitative assays, for the first time. Through these assays we have convincingly demonstrated for the first time, multiple conserved features of apoptosis in embryonic stages of a pathogenic filarial nematode. Further, in the present study we have demonstrated characteristic features of apoptosis e.g. mitochondrial depolarization, redistribution of Cytochrome-c, formation of hypodiploid nuclei etc. which has not been demonstrated in the model nematode *C.elegans*, so far. We have gone one step ahead to evaluate the effect of some of the existing pharmacological compounds for their apoptogenicity which has opened up avenues for development of embryogenesis blocking drugs against nematode pathogens. Finally, the induction of apoptosis in embryonic stages by lipid peroxidation product molecules, commonly associated with inflammatory responses in filarial disease [19] and demonstration of in-situ apoptosis of developing embryos in adult female parasites in a natural bovine model of filariasis [20] have offered a framework to understand anti fecundity host immunity against parasitic worms.

## Methods

### Ethics statement

No investigations were undertaken using humans/human samples in this study. No experimental animals were used to conduct any of the experiments reported in this manuscript. Adult *Setaria digitata* worms from peritoneal cavities and blood samples from slaughtered cattle were collected from the abattoir attached to a local zoo after obtaining approval from zoo authorities. The animals are slaughtered in the abattoir regularly for feeding the wild cats and no animals were slaughtered specifically for the purpose of our study.

### Materials

Hanks Balanced Salt Solution [HBSS] medium, RPMI-1640 medium, glucose, Penicillin, Streptomycin, Amphotericin – B, pancaspase inhibitor Z-VAD–FMK [N-Benzyloxycarbonyl- Val-Ala-Asp[O-Me] fluromethyl ketone], Colorimetric caspase substrate Ac-DEVD-pNA [N-Acetyl- Asp-Glu-Val-Asp p-nitroanilide], Fluorogenic substrate for reactive oxygen species H2-DCFDA[2′–7′-Dichloro dihydro fluorescein diacetate] and anti- goat IgG –FITC antibodies were purchased from Sigma. Annexin-V- PE apoptosis detection kit-I, Mitoscreen JC-1 kit, Apo-Direct apoptosis detection kit and fluorochrome conjugated antibodies for cleaved PARP (Poly [ADP-ribose] polymerase) were purchased from BD Biosciences. Fluorochrome conjugated antibodies to Cytochrome-c was procured from eBioscience. Primary goat antibodies to CED-3, CED-4 and CED-9 and anti-goat IgG-PE were purchased from Santacruz. Mito Tracker red CMX Ros was purchased from Invitrogen Molecular Probes. Lipid Peroxidation Products were purchased from Sigma Aldrich.

### Isolation of developing embryos of *S.digitata*

Adult female filarial worms *Setaria digitata* were collected from the peritoneum of cattle, slaughtered at a nearby abattoir and transported to the laboratory in sterile Hanks Balanced Salt Solution [HBSS] medium [Sigma H 2387]. The medium containing 1% glucose [Sigma G 7525], Penicillin 100 units/ml, Streptomycin 100 µg/ml [Sigma P 4333], and Amphotericin - B 0.25 µg/ml [Sigma A2942] was buffered with NaHCO_3_ [Sigma S 5761]_._ About 5 to 7 worms were taken in a petridish, washed three times in medium, dissected into small pieces in 10 ml of medium under sterile conditions and incubated at 37°C for 30 minutes to allow the release of embryonic stages [eggs and microfilariae] into the medium. The embryonic stages were harvested into sterile 15 ml centrifuge tubes and washed three times by centrifuging at 300 g for 10 minutes each with medium and the final pellet was suspended in 1 ml of RPMI-1640 medium [Sigma R 8005] supplemented with 10% FBS [Sigma F 2442].

### In-vitro culture and treatment of embryonic stages

One ml each from a suspension containing 1×10^5^ embryonic stages/ml was dispensed into individual wells of a 24 well tissue culture plate. Control set of wells were left untreated. Other sets of wells containing suspension of developing embryos were subjected to treatment with various agents separately at a concentration ranging from 10–100 µM for 24–48 hr. at 37°C in a 5% CO_2_ incubator. Motility of microfilariae as a marker of viability of embryonic stages, after treatment with different agents was scored under an inverted microscope **[Video S1, S2 and S3].** Viability of embryonic stages was also checked by propidium iodide staining. In the present study 3 replicates of each treatment type were used except for the lipid peroxidation products for which 5 replicates each were used.

### Flow cytometry

Following treatment and incubation, embryonic stages of *S.digitata* were analyzed either live or after fixation with 1% paraformaldehyde in a flow cytometer [BD FACS Calibur, Becten and Dickinson, USA]. Apoptosis of different developmental stages were studied by gating the respective population in the Dot Plots. Embryonic stages were stained for externalization of phosphatidylserine, mitochondrial depolarization and fragmentation of chromosomal DNA using Annexin-V: PE apoptosis detection kit-I [BD Biosciences 559763**]**, Mitoscreen JC-1 kit [BD Biosciences 551302], Apo-Direct apoptosis detection kit [BD Biosciences 556381] respectively, as per the manufacturer's instructions. Intra cellular profile of apoptosis related proteins was studied using either fluorochrome conjugated antibodies directly viz,. anti-Cytochrome c-FITC [e Bioscience 11-6601-82]**/** anti cleaved PARP–PE [BD Biosciences 552933], or primary goat antibodies [1: 100 diluted] such as anti CED-3 [Santacruz sc- 9192**]** /anti CED-4 [Santacruz sc-9193**]** /anti CED-9 [Santacruz sc- 9202] followed by probing with fluorochrome conjugated secondary antibodies [1∶100 diluted] viz., anti-goat IgG-PE [Santacruz sc-3747]/ anti- goat IgG -FITC [Sigma F7367]. Incubation for both primary and secondary antibodies was performed for 1 hr at 37°C in dark. Analysis was performed on 10,000 acquired events in flow cytometer using Cell quest Pro software.

### Immunofluorescence microscopy

Subcellular distribution and colocalization profile of Cytochrome-c and CED-4 were studied by analyzing the immuno-stained embryonic stages under confocal microscope [Leica, Germany] equipped with a 63X objective after mounting them in 30% glycerol on glass slides. Sub cellular localization of CED-4 in developing embryos was confirmed by colocalization experiments with Mito Tracker red CMX Ros [Invitrogen Molecular Probes M 7512], used at a final concentration of 50 nM [incubation was done for 45 minutes on ice].

### Confirmation of the caspase activity

Activity of caspase family of cysteine proteases was confirmed by detecting the presence of cleaved PARP through inta cellular staining, using fluorochrome conjugated antibodies to cleaved PARP [BD Biosciences 552933], studying the inhibition of externalization of phosphatidylserine using the cell permeable pan caspase inhibitor Z-VAD–FMK [Sigma V 116], used at a concentration of 50 µM [preincubation was done 1 hr before treatment] by flow cytometry and a colorimetric assay by incubating the cell lysate of the in-vitro cultured and treated embryonic stages with the colorimetric Caspase substrate Ac-DEVD-pNA [Sigma A 2559] for 2 hrs at 37°C at a final concentration of 200 µM.

### Detection of intra cellular ROS (Reactive Oxygen Species)

The intracellular accumulation of ROS was measured in terms of DCF fluorescence by using the fluorescent probe [2, 7, Dichloro Dihydro Fluorescein Diacetate] H_2_-DCFDA as described previously [21], [22] with little modification. Briefly, the cultured developing embryos of *S.digitata* were pre incubated with 5 µM H_2_-DCFDA [Sigma D 6883] before treatment with different pharmacological agents mentioned earlier. The control set of embryonic stages were also subjected to the same manipulation except for treatment with any of the agents. After 24 hrs of incubation, the developing embryos were harvested washed with PBS twice and analyzed by flow cytometer. Inhibition of intracellular ROS was studied using antioxidant N-Acetyl-L-Cysteine [Sigma A 7250].

### Homology modeling and molecular docking

The amino acid sequence of Cytochrome-c of *C. elegans* [target] was retrieved from the sequence database of NCBI [P19974] and its 3-D structure was generated by homology modeling, using the academic version of MODELLER9v6 software [23]. The crystal structure of Cytochrome-c from bovine heart [PDB code: 2B4Z] [24] which shares sequence identity and similarity of 61% and 74% respectively, with the target was taken as a template for the modeling. Out of 20 models generated by MODELLER, one with the best G-score of PROCHECK [25] and VERIFY -3 D [26] profiles was selected and subjected to energy minimization using CHARMM [27] force field. The final stable 3-D structure of Cytochrome-c of *C.elegans* was used for molecular docking with CED-4, using Patch Dock algorithm. The top 20 solutions, out of about 60 predicted Cytochrome-c-CED4 complexes were sorted according to their geometric shape, followed by refinement and ranking of the models using Fire Dock server. The Fire Dock top rank was sent to PDB sum server to predict the protein-protein interactions. We have also retrieved the amino acid sequence of Cytochrome-c of human filarial parasite Brugia malayi [target] from the sequence database of NCBI [Accession NO. XP_001897096] and generated it's 3-D structure by homology modeling, using the academic version of MODELLER9v6 software [23] as described above. 1CCR was taken as a template for the modeling.

### Demonstration of induction of apoptosis of embryonic stages in-vivo

To ascertain whether apoptogenic compounds present in the external milieu surrounding live worms can exert their effect on embryonic stages present in the uterine cavity of intact adult female parasites, we designed a simulated experiment where we incubated, randomly selected adult female worms of comparable size [4 each] in two different 50ml sterile vials containing RPMI-1640 medium with 10% FBS. One set of worms was treated with 10 µM of Plumbagin while the other set was left untreated and regarded as control. After overnight incubation, developing embryos harvested separately from each set of cultured worms was subjected to ex-vivo analysis for apoptosis in terms of expression of caspase homologue, CED-3; cleavage of intracellular caspase substrate PARP; and fragmentation of chromosomal DNA.

### Demonstration of in-situ apoptosis of developing embryos

Female adult worms were collected from the peritoneum of infected cattle, slaughtered in a nearby abattoir. Along with worms, 10 ml of blood was also collected in separate sterile acid citrate dextrose [ACD] vial from each animal. After lysing the RBCs in blood by distilled water washing, the final cell pellet was resuspended in TEBS buffer and passed through 5 µM polycarbonate membrane [Nucleopore, USA]. The membrane filter was then checked for retention of microfilariae under light microscope. The result was further confirmed by Giemsa stained thick blood smears. Depending upon the presence or absence of microfilariae, the naturally infected bovine hosts were categorized into microfilaraemic and amicrofilaraemic groups. The suspension of developing embryos harvested from adult female worms of each animal was divided into two fractions, followed by staining of one fraction with dUTP – FITC in the presence of TdT enzyme[experimental fraction] and the other fraction with dUTP – FITC in the absence of TdT enzyme[control fraction] ex-vivo. The GMI(Geometric mean intensity) of dUTP-FITC fluorescence for endogenous fragmentation of DNA as a measure of apoptosis in the developing embryos was obtained after subtracting the GMI of dUTP-FITC fluorescence of control fraction [representing back ground fluorescence] from that of experimental fraction. Apoptosis in terms of extent of DNA fragmentation in microfilariae, early and late embryonic stages was scored after gating respective population in the Dot Plots as described earlier in this study.

### Statistical analysis

Paired comparison were conducted using paired t – test, and all data are presented as mean value ± SEM. Differences were considered significant at 95% confidence levels. All statistical analysis was performed with Graph Pad Prism, version 5.0.

## Results

### Apoptosis inducing agents precipitate conserved cytoplasmic and nuclear features of apoptosis in developing embryos of a pathogenic nematode

**Figure 1:**
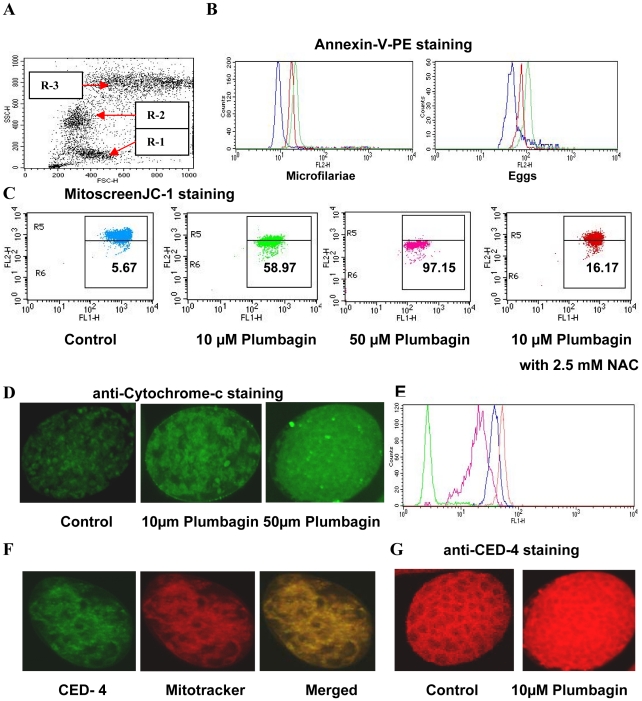
Demonstration of characteristic membrane and cytoplasmic features of apoptosis in developing embryos of *S.digitata*. Developing embryos harvested from adult *S.digitata* worms were analyzed with a flow cytometer [BD FACS Calibur] using Dot plots and Histogram plots. [**A**] Dot plots for embryonic stages showing 3 distinct clusters of populations: R-1 representing microfilariae while R-2 and R-3 representing eggs-early and late embryonic stages respectively. Apoptosis was studied with different assays either by gating respective populations in the Dot plots – for 3 different populations of embryonic stages, individually or without gating - for the embryonic stages all together. [**B**] Overlaid histograms show phosphatidyl serine exposure on microfilariae and eggs by Annexin-V-PE staining after treatment with 10 µM [**Red**] or 50 µM**[Green]** of Plumbagin in comparison to untreated controls**[Blue]** [**C**] Dot Plots revealing depolarization of mitochondria in embryonic stages and its reversal by NAC are shown using Mitoscreen JC-1 staining. The percentage of events in the upper gate [R5] and lower gate [R6] represent population of embryonic stages having normal and depolarized mitochondria respectively. [**D**] Confocal microscopic images of untreated embryos showing punctate staining and Plumbagin treated embryos showing diffusely cytoplasmic staining for Cytochrome-c respectively. [**E**] Overlaid histogram shows increased cytosolic presence of Cytochrome-c in developing embryos treated with 10 µM **[Pink]**, 50 µM **[Blue]** and 100 µM **[Red]** of Plumbagin over untreated control **[Green]**. [**F**] Confocal microscopic images of untreated normal embryos demonstrating colocalization of CED-4 with Mito Tracker Red are shown [**G**] Confocal microscopic images of untreated and Plumbagin treated embryos revealing web like cytoplasmic staining [similar to Mito Tracker Red] and diffusely cytoplasmic staining for CED - 4 respectively, are shown.

In the absence of suitable quantitative assay systems to score apoptosis in nematode embryos we developed flow cytometry based assays to initially demonstrate multiple conserved features of apoptosis in developing embryos of *S.digitata* [Table 1]. Apoptosis of developing embryos, which includes microfilariae /larval stage-1 and eggs, was scored by gating the respective populations [Figure 1A]. The identity of microfilariae and egg populations in dot plots for developing embryos of *S.digitata* was previously reported by us [28]. After induction of apoptosis using a known apoptogenic compound Plumbagin [29], [30] surface and intracellular staining of developing embryos were performed to demonstrate conserved apoptotic features. Plumbagin induces intracellular ROS mediated mitochondrial pathway of apoptosis in different cellular systems [29], [30]. Since we are addressing intrinsic pathway of apoptosis in developing embryos of the filarial nematode, we chose to use Plumbagin in this study.

**Table 1:** GMI [Geometric Mean Intensity] of fluorescence for each of the 8 assays for apoptosis in developing embryos of pathogenic nematode *S.digitata* after Plumbagin treatment.

Assay*	Microfilariae/Larval stage-1			Eggs/Prelarval embryonic stages		
	Control	10 µM	50 µM	Control	10 µM	50 µM
Annexin-V	7.52	12.67	22.56	27.48	85.27	105.49
CED-3	7.97	33.91	41.70	94.08	204.26	218.97
CED-4	8.42	35.87	41.54	95.91	211.21	226.90
CED-9	10.71	32.16	37.64	108.38	204.81	214.76
Cytochrome-c	4.52	18.85	38.32	57.36	72.62	88.71
CleavedPARP	7.55	10.33	19.50	65.57	107.25	120.64
TUNEL	10.52	41.79	81.74	66.08	106.44	124.19
JC-1**	983.84	420.03	256.68	1530.26	982.16	554.77

*The values in the table represent the mean of GMI of fluorescence of 3 independent experiments for each assay.

**Mitochondrial Depolarization assay by Mitoscreen-JC-1-Staining- expressed in terms of reduction in GMI of fluorescence.

**Figure 2:**
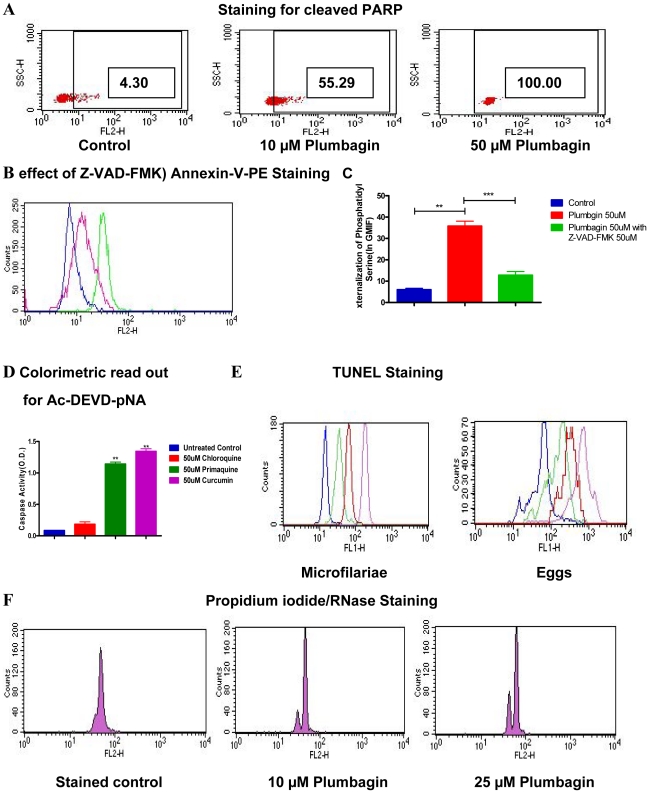
Demonstration of caspase activity and nuclear features of apoptosis in developing embryos of *S.digitata.* [**A**] Dot Plots revealing dose dependent increase in cleavage of intra cellular caspase substrate PARP [Poly [ADP-ribose] polymerase] in embryonic stages, treated with Plumbagin is shown. In Dot Plots, percentage of population shifting right into the gate after Plumbagin treatment [in comparison to untreated controls] represents embryonic stages showing PARP cleavage. [**B**] Overlaid histograms demonstrating externalization of phosphatidyl serine with 50 µM Plumbagin **[Green]** and its inhibition with [50 µM] pan-caspase inhibitor Z-VAD–FMK [N-Benzyloxycarbonyl-Val-Ala-Asp [O-Me] fluromethyl ketone][**Pink]** over untreated control **[Blue]** is shown. [**C**] Externalization of Phosphatidyl serine and it's inhibition in embryonic stages treated with Plumbagin for 24 hr in the presence or absence of Z-VAD-FMK is shown. Data points in C represent mean ± SEM, with *n* = 3. **P<0.05 by Paired t-test; ***P<0.05 by Paired t-test. [**D**] Colorimetric assay revealing Caspase activity in embryonic stages using colorimetric caspase substrate - Ac-DEVD-pNA [N-Acetyl-Asp-Glu-Val-Asp p-nitroanilide] is shown. Data points in D represent mean ± SEM, with *n* = 3. **P<0.05 by Paired t-test versus untreated controls. [**E**] Overlaid histograms show fragmentation of chromosomal DNA in embryonic stages upon treatment with 10 µM **[Green]**, 50 µM **[Red]** or 100 µM **[Violet]** of Plumbagin in comparison to untreated controls **[Blue]** [**F**] Histogram plots demonstrating formation of hypo-diploid nuclei [represented by a smaller peak with reduced fluorescence behind the normal peak] in Plumbagin treated developing embryos after PI/RNase staining are shown.

Annexin-V-PE staining revealed dose dependent externalization of phosphatidyl serine [Figure 1B and Table 1] and Mitoscreen JC-1 staining demonstrated mitochondrial depolarization [Figure 1C and Table 1] in embryonic stages undergoing apoptosis. The diffusely cytoplasmic staining pattern for evolutionarily conserved molecule - Cytochrome-c in apoptotic embryos as opposed to punctate staining in normal embryos [Figure 1D] and overlaid histogram for intra cellular staining of Cytochrome-c [Figure 1E and Table 1] suggested redistribution of Cytochrome-c during apoptosis. Enhanced intracellular expression of nematode apoptosis related proteins CED-3, CED-4 and CED-9 [Table 1] in embryonic stages of *S.digitata* were also observed upon stimulation with Plumbagin. Intracellular staining with antibodies to CED-4 revealed a distinct web like staining pattern similar to that of Mitotracker Red which specifically labels mitochondria [Figure 1F] in untreated normal embryos indicating mitochondrial localization of CED-4. Whereas, diffusely cytoplasmic staining pattern of CED-4 in apoptotic embryos of *S.digitata* [Figure 1G] suggests redistribution of CED-4 in response to apoptotic stimuli. These observations for CED-4 in apoptotic embryos of pathogenic nematode *S.digitata* were comparable with observations made with apoptotic embryos of free living nematode *C.elegans* [31]. Intracellular staining with fluorochrome conjugated antibodies to cleaved PARP demonstrated cleavage of highly conserved intra cellular caspase substrate-PARP [Figure 2A and Table 1] indicating activation of caspase family of cysteine proteases during apoptosis in developing embryos of *S.digitata*. Further, inhibition of apoptosis by pan caspase inhibitor, Z-VAD – FMK [Figure 2B and C] and quantitative assay for caspase activity using colorimetric caspase substrate Ac-DEVD-pNA [Figure 2D] corroborated the role of caspase activity in embryonic stages during apoptosis. Analysis of nuclear features of apoptosis revealed fragmentation of chromosomal DNA as shown by TUNEL assay [Figure 2E and Table 1] and formation of sub diploid nuclei by PI [propidium iodide]/RNase staining [Figure 2F] in developing embryos of *S.digitata.* Taken together, these results represent the first ever definitive demonstration of conserved cytoplasmic and nuclear features of apoptosis in a pathogenic nematode.

### Screening of pharmacological agents and Lipid Peroxidation Products for apoptogenicity against developing embryos

**Figure 3:**
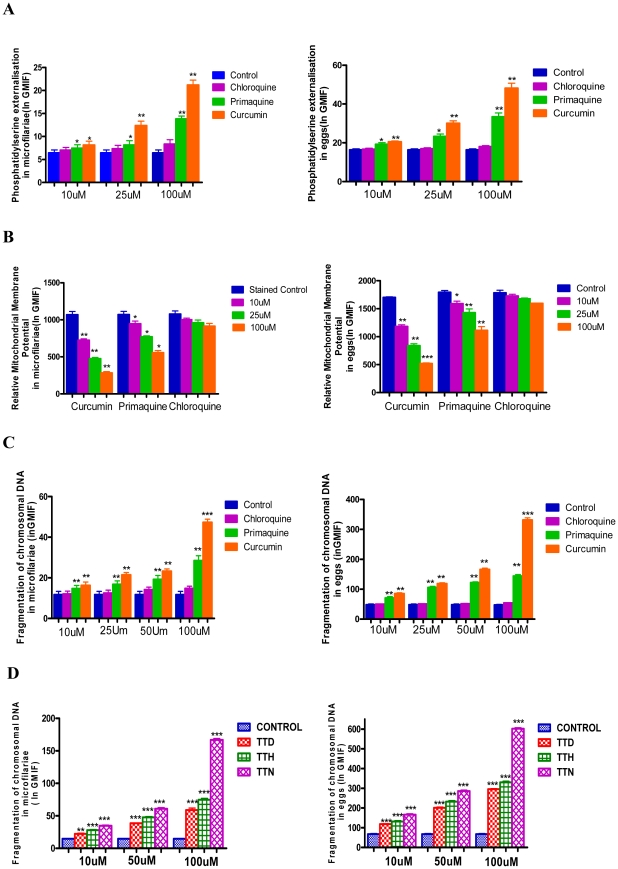
Comparison of apoptogenicity of 3 pharmacological agents and 3 LPPs (Lipid Peroxidation Products). [**A**],[**B**],[**C**]. Quantitation of externalization of phosphatidyl serine [**A**]; mitochondrial membrane potential [**B**] and fragmentation of chromosomal DNA[**C**] in embryonic stages, treated with pharmacological agents viz. Curcumin, Primaquine and Chloroquine for 48 hrs are shown. Data points in A, B, C represent mean ± SEM, with *n* = 3. *P<0.05 by Paired t-test versus untreated controls; **P<0.05 by Paired t-test versus untreated controls and *** P<0.05 by Paired t-test versus untreated controls [**D**] Quantitation of fragmentation of chromosomal DNA in embryonic stages, treated with LPPs for 48 hrs. Data points in **D** represent mean ± SEM, with *n* = 5.*** P<0.05 by Paired t-test versus untreated controls.

Following demonstration of canonical features characteristic of apoptosis using Plumbagin, we evaluated apoptogenicity of some pharmacological agents viz., Curcumin, Primaquine, Chloroquine and Diethylcarbamazine [DEC] and three lipid peroxidation products [LPPs] namely TTH [Trans-Trans 2-4 Heptadienal], TTN [Trans-Trans 2-4 Nonadienal] and TTD [Trans-Trans-2–4 Decadienal] against developing embryos of *S.digitata*. Curcumin, which activates ROS mediated apoptosis in mammalian systems [32] was found to be the most effective pharmacological agent followed by Primaquine as shown by externalization of phosphatidyl serine, mitochondrial depolarization and fragmentation of chromosomal DNA while Chloroquine displayed minimal effect [Figure 3 A–C] and DEC had no demonstrable effect [Data not shown]. Amongst lipid peroxidation products TTN was found to be very efficient in inducing apoptosis of embryonic stages followed by TTH and TTD as shown by TUNEL assay [Figure 3 D].

### Intra cellular ROS plays a key role in mediating apoptosis in developing embryos

**Figure 4:**
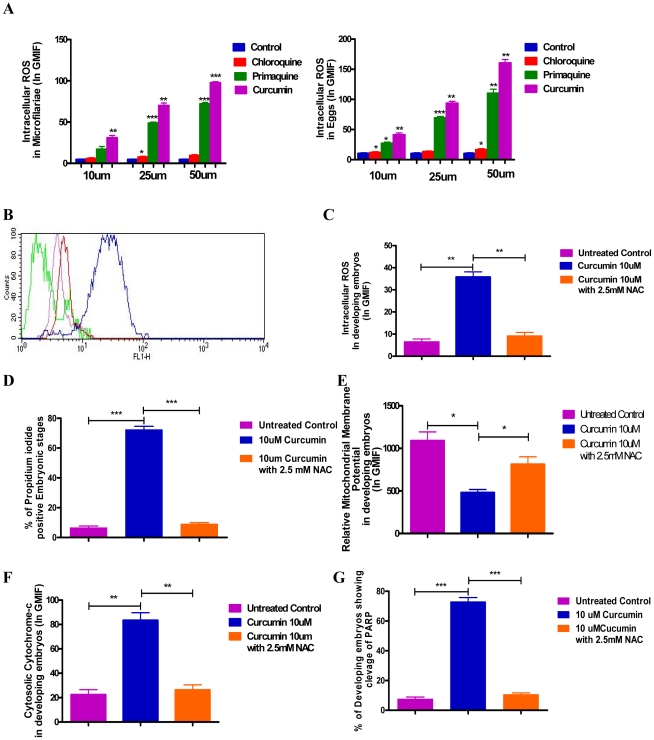
Role of intra cellular ROS, during apoptosis in developing embryos of *S.* *digitata.* [**A**] Quantitation of intra cellular ROS generated in embryonic stages after 24 hr treatment with pharmacological agents in terms of relative DCF [Dichloro fluorescein] fluorescence. Data points in A represent mean ± SEM, with *n* = 3. *P<0.05 by Paired t-test versus untreated controls; **P<0.05 by Paired t-test versus untreated controls;*** P<0.05 by Paired t-test versus untreated controls [**B**] Overlaid histogram revealing inhibition of intra cellular ROS by pre-treatment with 2.5mM [**Red**] and 5 mM [**Violet**] concentrations of NAC [N-Acetyl-L-Cystiene] - a scavenger of intracellular ROS in embryonic stages, treated with 10 µM of Curcumin [**Blue**] is shown. [**C**],[**D**],[**E**],[**F**],[**G**] Quantitation of intra cellular ROS [**C**]; viability [**D**], mitochondrial membrane potential [**E**]; cytosolic Cytochrome-c [**F**] and cleavage of intracellular caspase substrate PARP in embryonic stages treated with Curcumin in presence or absence of 2.5 mM NAC are shown. Data points in **C, D, E, F** and **G** represent mean ± SEM, with *n* = 3. *P<0.05 by Paired t-test; **P<0.05 by Paired t-test and *** P<0.05 by Paired t-test.

Several proapoptotic stimuli including ROS are known to mediate MOMP [Mitochondrial outer membrane permeabilization] and release Cytochrome-c from IMS [Inter membrane space] of mitochondria leading to apoptosis in mammalian systems [33]. Screening of pharmacological agents in the present study revealed that Curcumin and Primaquine induce intra cellular ROS [Figure 4A] in embryonic stages similar to their potential to induce externalization of phosphatidyl serine, mitochondrial depolarization and fragmentation of chromosomal DNA [Figure 3 A-C] i.e. induction of ROS matched with degree of apoptosis mediated by Curcumin and Primaquine. Further, N-Acetyl-L-Cysteine [NAC], a known scavenger of ROS [21], [22] not only inhibited ROS generation [Figure 4B,C] but also reversed apoptotic death of developing embryos, as shown by significant decrease in propidium iodide positivity among embryonic stages [Figure 4 D], reversal of several features of apoptosis [e.g. mitochondrial depolarization [Figure 1C and Figure 4E], redistribution of Cytochrome-c [Figure 4F] and PARP cleavage [Figure 4G]] and restoration of microfilarial motility [Video S1, S2 and S3] scored by microscopy [data not shown]. Taken together, these results implicate ROS in mediating apoptosis of developing embryos in this study and suggest possible existence of mitochondrial cell death pathway in parasitic worms.

### Redistribution of Cytochrome-c occurs after induction of apoptosis in developing embryos

**Figure 5:**
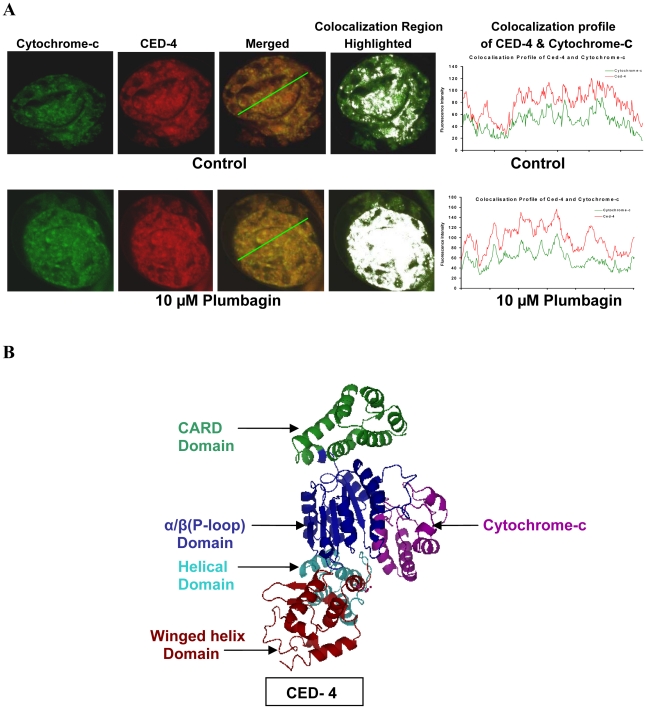
Demonstration of Cytochrome-c-CED-4 interaction in *S.digitata* and docking of Cytochrome-c and CED-4 of *C.elegans.* [**A**] Confocal images of untreated control [upper panel] or Plumbagin treated [lower panel] late embryonic stages demonstrating enhanced colocalization of Cytochrome-c and CED-4 after 24 hr Plumbagin treatment are shown. Regions of colocalization are highlighted as white patches using Image J software. Fluorescence intensity line profile/colocalization profile analysis for both the labeled proteins-Cytochrome-c and CED-4 in control as well as apoptotic embryos, revealing enhanced cololocalization of these proteins in the later are shown [**B**] Interaction of Cytochrome-c of *C.elegans* [*Magenta*] with α/β [P-loop] ATP binding domain [*Blue*] of CED-4 is shown.

Cytosolic release of Cytochrome-c and its role in caspase activation during apoptosis is yet to be demonstrated in nematodes [34]–[37]. Hence, we examined the status of Cytochrome-c during apoptosis in developing embryos of *S.digitata.* Observations in the present study have revealed redistribution of Cytochrome-c during caspase dependent apoptotic death in developing embryos [Figure 1D and Table 1]. These findings indirectly suggest a possible role of Cytochrome-c during caspase activation in developing embryos of *S.digitata*. In mammalian system of apoptosis, caspase activation usually involves cytosolic release of Cytochrome-c and its subsequent interaction with Apaf-1 leading to formation of apoptosome [35]. However, it is not known whether similar interaction between Cytochrome-c and Apaf-1 homologue-CED-4 occurs in nematode system of apoptosis. Hence, demonstration of redistribution of Cytochrome-c and CED-4 in this study [described earlier] was followed by analysis of the sub cellular localization of these two proteins in apoptotic embryos of *S.digitata*. Results of immuno-localization study revealed significant degree of colocalization between CED-4 and Cytochrome-c in Plumbagin treated apoptotic embryos [Figure 5A] suggesting a possible interaction between these two proteins during activation of apoptosis. This finding was further confirmed by performing fluorescence intensity line profile/colocalization profile analysis for both the labeled proteins in control as well as apoptotic embryos [Figure 5A]. The only nematode for which complete genome data base is available is *C.elegans.* Hence, to substantiate our experimental finding, molecular docking studies between CED-4 and Cytochrome-c was undertaken, using protein data base of *C.elegans.* Since the crystal structure for Cytochrome-c of *C.elegans* was not available, homology modeling for three dimensional structure of Cytochrome-c followed by molecular docking with CED-4 of *C.elegans* was performed [Figure 5B, Figure S1 and S2] which revealed significant interaction between these two proteins of *C.elegans* involving 5 hydrogen bonds and 262 hydrophobic interactions [Figure S2]. The Cytochrome-c molecules are known to be highly conserved. The molecular docking of *Brugia malayi* Cytochrome-c with CED-4 also revealed significant interaction between these two molecules involving 11 hydrogen bonds and 232 hydrophobic interactions [Figure S3]. In mammalian systems, Cytochrome-c has been reported to enhance binding as well as hydrolysis of dATP by Apaf-1 during apoptosome formation [38], [39]. Hence, the predicted interaction of Cytochrome-c with the evolutionarily conserved α/β [P-loop] – ATP binding domain of CED-4, that leaves the CARD domain of the later, free for interaction with its target proteins [Figure 5B] suggests that such interactions between Cytochrome-c and CED-4 could be functionally relevant during activation of caspase homologue - CED-3 in nematodes. However, the above observations provide only preliminary evidence for possible role of Cytochrome-c in nematode system of apoptosis and further studies are needed to establish the precise role of Cytochrome-c in the process.

### Treatment of live adult worms with apoptogenic compounds mediates apoptosis of developing embryos of a pathogenic nematode in vivo

**Figure 6:**
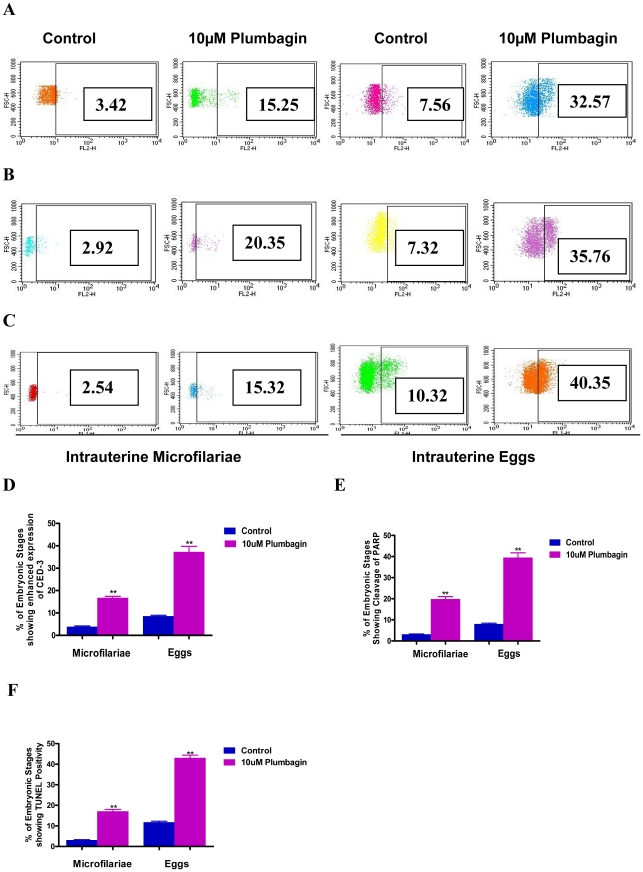
Treatment of intact adult worms with Plumbagin induces apoptosis of developing embryos in vivo. [**A**],[**B**],[**C**] Female adult worms were treated overnight with 10 µM of Plumbagin after which developing embryos were harvested, fixed with 1%para formaldehyde for 1 hr at 0°C, and stained for apoptosis – enhanced expression of CED-3[**A**]; cleavage of intra cellular caspase substrate PARP [**B**] and fragmentation of chromosomal DNA [**C**] were detected. In Dot Plots, percentage of population shifting right into the gate after Plumbagin treatment [in comparison to untreated controls] represents apoptotic embryonic stages. Data points in **D, E** and **F** represent mean ± SEM, with *n* = 3. **P<0.05 by Paired t-test versus untreated controls.

The screening experiments with pharmacological agents and LPPs in this study were performed by treatment of developing embryos, released from adult female worms in vitro. To simulate a physiological scenario, intact live adult worms were treated with Plumbagin followed by analysis of their intra uterine embryonic stages for apoptosis ex vivo. Three independent sets of experiments yielded comparable results - the embryonic stages from the adult female parasites treated with Plumbagin revealed significantly higher degree of apoptosis in terms of expression of CED-3 [Figure 6A and D], cleavage of caspase substrate PARP **[** Figure 6B and E **]** and fragmentation of chromosomal DNA [Figure 6C and F]. These observations suggests that the phenomenon of exogenous induction of apoptosis in developing embryos of nematode worms could be biologically relevant and that apoptosis inducing pharmacological agents could be used for blocking embryogenesis in adult female parasites in infected hosts. Similarly, host effector molecules such as lipid peroxidation products present in circulation of an infected host could potentially exert their anti-fecundity effects by induction of apoptosis in developing embryos in vivo.

### Enhanced in-situ apoptosis of embryonic stages in adult filarial worms collected from amicrofilaraemic animals in a bovine model of filariasis

**Figure 7:**
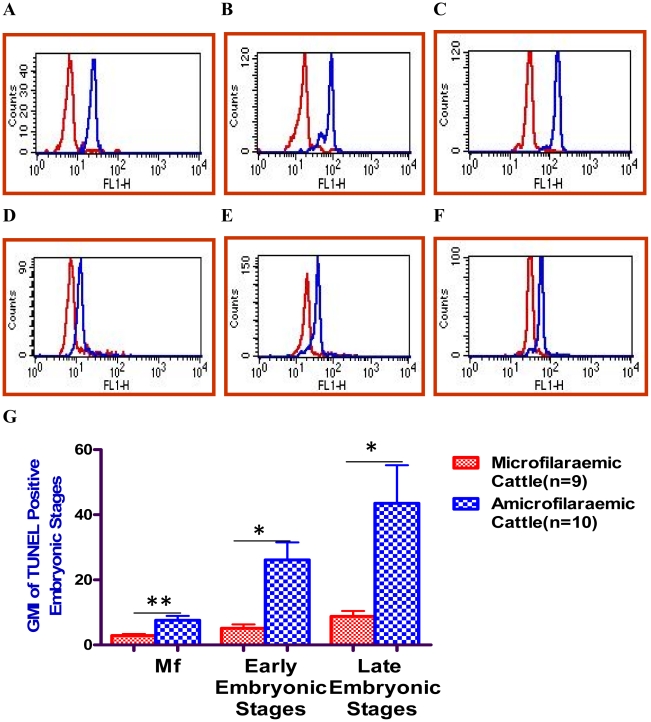
Demonstration of in-situ apoptosis of embryonic stages of *S. digitata* by TUNEL staining. Embryonic stages harvested from adult female worms, collected from the peritoneum of amicrofilaraemic and microfilaraemic cattle were subjected to TUNEL staining ex-vivo. Depending upon the presence or absence of microfilariae in the blood [determined as described in the methods section], the naturally infected bovine hosts were first categorized into microfilaraemic and amicrofilaraemic groups. The suspension of developing embryos harvested from adult female worms of each host animal was divided into two fractions, followed by staining of one fraction with dUTP – FITC in the presence of TdT enzyme[experimental fraction] and the other fraction with dUTP – FITC in the absence of TdT enzyme [control fraction] ex-vivo. The GMI of dUTP-FITC fluorescence for endogenous fragmentation of DNA as a measure of apoptosis in the developing embryos was obtained after subtracting the GMI of dUTP-FITC fluorescence of control fraction [representing back ground fluorescence] from that of experimental fraction. Apoptosis in terms of extent of DNA fragmentation in microfilariae[**A,D**], early[**B,E**] and late embryonic stages[**C,F**] was scored after gating respective populations in the Dot Plots as described earlier in this study. Data points in the Figure [**G**] represent Mean ± SEM, with n = 9 for microfilaraemic cattle and n = 10 for amicrofilaraemic cattle. *P<0.05 by Paired t-test and **P<0.05 by Paired t-test.

Anti-fecundity immunity in lymphatic filariasis is characterized by presence of adult worms in host in the absence of circulating microfilariae. The mechanism by which human as well as bovine hosts achieves this status in filariasis has not been studied so far [15]. The possibility that enhanced apoptosis of developing embryos during embryogenesis could lead to a state of amicrofilaraemia, in hosts harboring gravid adult female worms was addressed in this study for the first time, in a bovine model of filariasis [20]. Endogenous apoptosis was quantified in developing embryos of *S.digitata,* harvested from naturally infected bovine hosts [20] with two different parasitological status [e.g. **i-** cattle harboring both adult worms in peritoneum and microfilariae in circulation and **ii**- amicrofilaraemic cattle with only adult worms in peritoneum but no circulating microfilariae] by TUNEL staining of embryonic stages ex-vivo. Enhanced apoptosis was found in the developing embryos of adult female parasites, isolated from second category of bovine hosts **[** Figure 7 **]** suggesting that increased apoptosis during embryogenesis [partly mediated by host factors], could be responsible for significantly less production and release of viable microfilariae by gravid adult worms into the peripheral circulation of host leading to their amicrofilaraemic status. These findings suggest, induction of apoptosis in developing embryos could be a novel effector mechanism underlying anti-fecundity host immunity operational in helminthiasis.

## Discussion

Apoptosis is a genetically controlled conserved mechanism of cellular demise that plays an important role in a wide variety of physiological processes including embryogenesis, tissue homeostasis and disease progression [40]. During host parasite interaction, parasites tend to induce apoptosis in host cells as a strategy of survival and as a means of establishing infection in their hosts, by creating a site of immune privilege around them [41]–[43]. Similarly, host cells may induce apoptosis of parasites as a defense strategy which has been well documented in case of unicellular parasites only [43]. However, unlike protozoan parasites, metazoan parasites are difficult targets for the cells of host immune system. The disproportionately large size of metazoan helminth parasites vis-a-vis the host immune effector cells and prolonged survival of the former inside respective mammalian hosts has led to a perception that protective immunity may not be effectively operational against these pathogens [15]. Hence, induction of apoptotic death as a therapeutic approach seems to be more relevant in case of these extra cellular parasites. On the other hand, successful embryogenesis in adult females is a critical rate limiting step for survival and propagation of parasitic worms as well as pathology mediated by them [18]. Therefore, blockade of embryogenesis through induction of apoptosis in early embryonic stages offers a viable alternative for developing intervention strategies against them. But, the potential of therapeutic induction of apoptosis as an approach for drug development against metazoan helminth parasites has not been explored, so far. Hence, in the present study we have made an attempt to venture into this unexplored area of research in the biology of parasitic worms.

Since, apoptosis in developing embryos of pathogenic nematodes has not been reported in literature earlier, we considered it essential to assess several conserved features of apoptosis including externalization of phosphatidyl serine, mitochondrial depolarization and activation of caspase family of cysteine proteases, fragmentation of chromosomal DNA and formation of hypo-diploid nuclei in embryonic stages of a filarial nematode *S.digitata.* We further validated the observed programmed cell death by using immuno fluorescence microscopy and scoring expression profile of nematode specific proteins related to apoptosis [e.g. CED-3, CED-4 and CED-9] in the embryonic stages. Studies in the free living nematode *C.elegans* had identified three nematode specific proteins such as CED-3, CED-4 and CED-9 associated with the process of apoptosis. Homolog of these three proteins has been shown to be involved in apoptosis in all most all systems studied so far [44]. The CED-3 protein is a member of the caspase family of cysteine proteases that executes the final cell death process. CED-4 protein is homologus to mammalian Apaf-1 that functions as a positive regulator of CED-3 whereas CED-9 protein is homologus to mammalian Bcl-2 and promotes cell survival in nematodes [45]. Further, redistribution of CED-4 could be demonstrated in apoptotic embryos of *S.digitata*, similar to earlier observations in apoptotic eggs of *C.elegans* [31]. Taken together, the above findings clearly suggest that the programmed cell death [PCD] observed in developing embryos of *S.digitata* in this study is a phenomenon of apoptosis.

Even though the size of adult nematodes varies greatly, their eggs are usually of comparable size [46]. In general, size of nematode eggs, which might contain a fully formed first stage larvae ranges from 30 µm to 100 µm in greatest diameter [47]. Since standard protocols are currently available for isolating eggs of parasitic worms [48] and the sample injection port of conventional flow cytometers including BD- FACS calibur used in this study ranges up to 150 µm, the quantitative flow cytometry based assays for apoptosis evaluated in this study are expected to be amenable to embryonic stages of other parasitic worms as well. Our earlier demonstration of the compatibility of first stage larvae/ microfilariae of human filarial parasite *Wuchereria bancrofti* to flow cytometry analysis [28] offers credence to our hypothesis.

Caspase activity is considered essential for general precipitation of typical nuclear features of apoptosis in mammalian cells. However, it is dispensable in several other instances for induction and execution of apoptosis [37], [49], [50]. In the present study we have convincingly demonstrated caspase activity in the apoptotic embryos of *S.digitata,* using multiple approaches [e.g. demonstrating cleavage of PARP - an evolutionarily conserved molecule targeted by active caspases, using assay for caspase activity with colorimetric caspase substrate Ac-DEVD-pNA, showing inhibition of phosphatidyl serine externalization by pan caspase inhibitor Z-VAD-FMK (N-Benzyloxycarbonyl-Val-Ala-Asp[O-Me] fluromethyl ketone) as well as studying intracellular expression profile of CED-3, a homologue of caspase in nematodes]. In typical conserved apoptotic pathways in mammalian cells, the effector caspases invariably lead to activation of a nuclease [- caspase activated DNAse/CAD, by cleavage of its inhibitor, ICAD -inhibitor of caspase activated DNase [50]] that is primarily responsible for fragmentation of chromosomal DNA and formation of sub diploid nuclei during apoptosis [51], [52]. Similar nuclear features of apoptosis were also observed in this study which corroborates activation of caspase family of cysteine proteases during induction of PCD in developing embryos in this study.

Mitochondria are regarded as an integrative organelle in terms of apoptosis as it acts as a meeting point of both caspase dependent and independent path ways of cell death [36]. It is also known as the major source and target of intracellular ROS [53]. Diversion of electrons from mitochondrial respiratory chain is the primary source of intracellular ROS [53] which in its turn acts back upon mitochondria to bring about its depolarization and trigger the release of pro apoptotic factors including Cytochrome-c in to cytosol. The cytosolic Cytochrome-c then interacts with Apaf-1 to form an apoptosome which ultimately lead to activation of effector caspase and apoptosis [51]. Thus reactive oxygen species [ROS] are considered to be essential mediators of apoptosis in various eukaryotic systems [53]. It has been reported that, molecules stimulating formation of ROS trigger apoptosis, a process that is inhibited in the presence of antioxidants [32]. In this context, observations in the current study viz., increased generation of ROS coupled with depolarization of mitochondria, redistribution of Cytochrome-c and cleavage of conserved intracellular caspase substrate PARP during induction of apoptotic death in developing embryos of filarial nematode *S.digitata* and reversal of all these features in presence of a known scavenger of ROS – NAC [21], [22] suggest a role for ROS in mediating apoptosis in this study and indicate possible existence of mitochondrial pathway of apoptosis in pathogenic nematodes. These findings also provide possibilities to design new strategies to kill nematode parasites preferentially through induction of ROS mediated programmed cell death.

Metazoan parasites elicit a unique mechanism of host immunity in their respective hosts commonly referred to as anti-fecundity immunity. This distinctive aspect of host immunity reduces output of viable embryos [e.g. eggs or microfilariae] by fecund female parasites into the peripheral circulation or tissues of infected hosts. However, the precise host mechanism underlying these anti-fecundity effects is yet to be established in literature. Taking into consideration the very high fecundity in parasitic worms, conventional methods to demonstrate anti-fecundity effects [e.g. measuring the circulating antigen; microscopic counting of the microfilariae in circulation or eggs in tissue homogenates, feces or urine of host and in uterine cavities of female worms by light microscopy etc. [54]-[56] do not offer reliable quantitative information on anti-fecundity effects of drugs or vaccines. In this context, the multiple quantitative flow cytometry based assays developed and evaluated in the present study for demonstration of apoptotic death in developing embryos of a filarial nematode, for the first time, represent a quantum improvement in our approach to understand the anti-fecundity effects mediated by drugs, vaccines and host immune cells or molecules in helminthic infections and are expected to find wide application in nematode biology. Further, induction of apoptotic death of developing embryos by LPPs observed in the present study; appears to be a possible effector mechanism of host against parasitic worms since, raised levels of plasma LPPs is an integral aspect of several parasitic diseases including helminth infections [19].

Usually cells far in excess are generated in metazoan organisms during early embryogenesis and many of them undergo apoptosis for embryogenic sculpturing of different tissues and organs in the late embryo [57]. In the present study the basal level of apoptosis was consistently found to be higher in pre larval embryonic stages as compared to microfilariae/larval stage-1 [L-1] [Table 1]. This quantitative difference in apoptosis among different developmental stages can be attributed to ongoing in-situ apoptosis during normal embryogenesis. Unlike pre larval embryonic stages which represent early stages of development in the filarial nematode *S. digitata,* microfilariae represent final stage of intra uterine development and hence are relatively free of ongoing in-situ apoptosis.

Infected humans in endemic areas can be classified into three groups based on presence of Circulating Filarial Antigen/CFA [products of adult worms] and microfilariae in circulation, namely endemic controls [neither CFA nor microfilariae in circulation], cryptic/amicrofilaraemic cases [with CFA but without microfilariae in circulation] and microfilariae carriers [with microfilariae in circulation] [15]. Several hypotheses have been proposed to explain the unusual cryptic/amicrofilaraemic status of human subjects with active filarial infection. Infestation with reproductively immature or unisexual worms, different anatomical location of male and female worms or anti microfilarial immunity [yet to be established in literature clearly] are some of the existing propositions in this regard [15]. The current study has used a bovine equivalent of this infection status observed in human filariasis [20]. Findings of the present study demonstrated enhanced in-situ apoptosis of developing embryos in adult female worms harvested from infected but amicrofilaraemic animals [**cryptic equivalent of human filariasis**] compared to that of microfilaraemic animals **[** Figure 7 **]** in a bovine model of filariasis. This observation provides preliminary evidence to suggest that anti-fecundity host immunity involving in-situ induction of apoptotic death of developing embryos [which can potentially reduce the output of viable microfilariae] can be another plausible explanation for the peculiar parasitological status observed in human lymphatic filariasis. However, further studies are needed to precisely establish the role of anti-fecundity host immunity in determining the clinical status of hosts in lymphatic filariasis.

In conclusion, findings of present study, constitute the first ever report on development and evaluation of flow cytometry based assays leading to clear demonstration of a common but hitherto unexplored phenomena i.e. apoptosis in developing embryos of a pathogenic nematode *S.digitata.* The observations in this study reveals that embryonic stages of metazoan filarial parasites are prone to caspase dependent apoptotic death, primarily mediated through induction of intra cellular ROS. Since apoptosis is a conserved biological process of cellular demise among metazoans, it's induction is expected to involve a closely similar mechanism, at least in a single group of animals i.e. among parasitic worms. Thus, compounds identified to have apoptogenicity towards one helminthic parasite/it's larval stages might prove effective against other helminthic pathogens, as well. In such an eventuality new drugs with broad spectrum anti helminthic activity with an established and common mode of action i.e. induction of apoptosis in embryonic stages will be a reality. The quantitative flow cytometry based assays for apoptosis evaluated in this study at a translational level, offers opportunities for developing automated high throughput screening assays for identification of anti-fecundity drugs and determining the efficacy of anti-fecundity vaccines to combat infections caused by helminth parasites. By providing a scope to understand the mechanism of an important phenomenon i.e. apoptosis in developing embryos of a pathogenic nematode, for the first time, the present study also offers leads to address other relevant issues of biological importance e.g. different forms of PCD, anti-fecundity host immunity, cell signaling and sex determination etc. during embryogenesis of parasitic worms. In addition, the present study can potentially further our understanding of genes that are critically important for embryo development and reproduction in parasitic worms, recently proposed to offer promise for developing alternative avenues of drug development against these metazoan parasites [8].

## Supporting Information

Figure S1 **3-D homology model of Cytochrome-c of** ***C.elegans.*** (A) Ramachandran plot of the modeled structure for Cytochrome-c of *C.elegans.* (B) Ribbon drawing of modeled Cytochrome-c of C.elegans. The amino acid sequence of Cytochrome-c of *C. elegans* (target) was retrieved from the sequence database of NCBI (P19974) and its 3-D structure was generated by homology modeling, using the academic version of MODELLER9v6 software. 2B4Z was taken as a template for the modeling.
(TIF)

Figure S2 **Predicted interactions between CED-4 and Cytochrome-c.** The molecular docking between CED-4 and Cytochrome-c revealed 5 hydrogen bonds (Blue lines) and 262 hydrophobic interactions (Orange lines) involving 33 residues of CED-4 and 25 residues of Cytochrome-c.
(TIF)

Figure S3 **(A) Ribbon drawing of modeled Cytochrome-c of** ***Brugia malayi*** **.** The amino acid sequence of Cytochrome-c of human filarial parasite Brugia malayi (target) was retrieved from the sequence database of NCBI (Accession NO. XP_001897096) and it's 3-D structure was generated by homology modeling, using the academic version of MODELLER9v6 software as described above. 1CCR was taken as a template for the modeling. (B) Interaction of Cytochrome-c of *Brugia malayi* with α/β (P-loop) ATP binding domain of CED-4 is shown.
(TIF)

Video S1 **Motility of Microfilariae of** ***S.digitata*** **in untreated control.**
(AVI)

Video S2 **Motility of Microfilariae of** ***S.digitata*** **after 6 hr treatment with 10 µm Plumbagin.**
(AVI)

Video S3 **Motility of Microfilariae of** ***S.digitata*** **after 24 hr treatment with 10 µm Plumbagin.**
(AVI)

## References

[1] Hotez PJ, Fenwick A, Savioli L, Molyneux DH (2009). Rescuing the bottom billion through control of neglected tropical diseases.. Lancet.

[2] Anthony RM, Rutitzky LI, Urban JF, Stadecker MJ, Gause WC (2007). Protective immune mechanisms in helminth infection.. Nat Rev Immunol.

[3] Maizels RM, Yazdanbakhsh M (2003). Immune regulation by helminth parasites: cellular and molecular mechanisms. Nat Rev Immunol..

[4] Gloeckner C, Garner AL, Mersha F, Oksov Y, Tricoche N (2010). Repositioning of an existing drug for the neglected tropical disease Onchocerciasis.. PNAS 107: 3424-.

[5] Hotez PJ, Molyneux DH, Fenwick A, Ottesen E, Ehrlich Sachs S (2006). Incorporating a rapid-impact package for neglected tropical diseases with programs for HIV/AIDS, Tuberculosis and malaria, PLoS Med..

[6] Song C, Gallup JM, Day TA, Bartholomay LC, Kimber MJ (2010). Development of an In Vivo RNAi Protocol to investigate Gene Function in the Filarial Nematode, *Brugia malayi*. Plos Pathog..

[7] Hotez PJ, Brindley PJ, Bethony JM, King CH, Pearce EJ (2008). Helminth infections: the great Neglected Tropical Diseases. J Clin Invest..

[8] Li BW, Rush AC, Jiang DJ, Mitreva M, Abubucker S (2011). Gender associated genes in filarial nematodes are important for reproduction and potential intervention targets PLoS Negl Trop Dis..

[9] Nwaka S, Hudson A (2006). Innovative lead discovery strategies for tropical diseases. Nat Rev Drug Discov..

[10] Bethony J, Brooker S, Albonico M, Geiger SM, Loukas A (2006). Soil-transmitted helminth infections: ascariasis, trichuriasis, and hookworm.. Lancet.

[11] James CE, Hudson AL, Davey MW (2009). Drug resistance mechanisms in helminths: is it survival of the fittest? Trends Parasitol..

[12] Geerts S, Gryseels B (2000). Drug resistance in human helminths: current situations and lesions from livestock. Clin Microbiol Rev.. 13: 207-.

[13] Bockarie MJ, Deb RM (2010). Elimination of lymphatic filariasis: do we have the drugs to complete the job? Curr Opin Infect Dis..

[14] Haarbrink M, Terhell AJ, Abadi GK, Mitsui Y, Yazdanbakhsh M (1999). Adverse reactions following diethylcarbamazine [DEC] intake in endemic normals, microfilaraemics and elephantiasis patients.. Trans R Soc Trop Med Hyg.

[15] Ravindran B, Satapathy AK, Sahoo PK, Mohanty MC (2003). Protective immunity in human lymphatic filariasis: problems and prospects.. Med Microbiol Immunol.

[16] Bruchhaus I, Roeder T, Rennenberg A, Heussler VT (2007). Protozoan parasites: programmed cell death as a mechanism of parasitism.. Trends Parasitol.

[17] Lee EF, Clarke OB, Evangelista M, Feng Z, Speed TP (2011). Discovery and molecular characterization of a Bcl-2-regulated cell death pathway in schistosomes.. Proc Natl Acad Sci USA.

[18] Freitas TC, Jung E, Pearce EJ (2007). TGF β signaling controls embryo development in the parasitic flatworms Schistosoma mansoni.. Plos Pathog.

[19] Pal BK, Kulkarni S, Bhandari Y, Ganesh BB, Goswami K (2006). Lymphatic filariasis: possible pathophysiological nexus with oxidative stress.. Trans R Soc Trop Med Hyg.

[20] Mohanty MC, Sahoo PK, Satapathy AK, Ravindran B (2000). *Setaria digitata* infections in cattle: parasite load, microfilaraemia status and relationship to immune response.. J Helminthol.

[21] Bensaad K, Cheung EC, Vousden KH (2009). Modulation of intracellular ROS levels by TIGAR controls autophagy.. EMBO J.

[22] Choi K, Ryu SW, Song S, Choi H, Kang SW (2010). Caspase-dependent generation of reactive oxygen species in human astrocytoma cells contributes to resistance to TRAIL-mediated apoptosis.. Cell Death Differ.

[23] Sali A, Blundell TL (1993). Comparative protein modeling by satisfaction of spatial restraints.. J Mol Biol.

[24] Mirkin N, Jaconcic J, Stojanoff V, Moreno A (2008). High resolution X-ray crystallographic structure of bovine heart cytochrome c and its application to the design of an electron transfer biosensor.. Proteins.

[25] Laskoswki RA, Mac Arthur MW, Moss DS, Thornton JM (1993). PROCHECK: a program to check the sterochemical quality of protein structures.. J Appl Cryst.

[26] Eisenberg D, Luthy R, Bowie JU (1997). VERIFY3D: Assessment of protein models with three-dimensional profiles.. Methods Enzymol.

[27] Brooks BR, Bruccoleri RE, Olafson BD, States DJ, Swaminathan S (1983). CHARMM: a program for macromolecular energy, minimization, and dynamics calculations.. J Comput Chem.

[28] Sahu BR, Mohapatra AD, Majumder A, Das PK, Ravindran B (2005). A flow cytometry based method for studying embryogenesis and immune reactivity to embryogenic stages in filarial parasites.. Filaria Journal.

[29] Wang CCC, Chiang YM, Sung SC, Hsu YL, Chang JK (2008). Plumbagin induces cell cycle arrest and apoptosis through reactive oxygen species / c-jun N-terminal kinase pathways in human melanoma A375.S2 cells.. Cancer Letters.

[30] Hsu YL, Cho CY, Kuo PL, Huang YT, Lin CC (2006). Plumbagin [5-Hydroxy-2- methyl-1, 4-napthoquinone] induces apoptosis and cell cycle arrest in A549 cells through p53 accumulation via c-jun NH-2-terminal kinase – mediated phosphorylation at serine15 in vitro and in vivo.. J Pharmacol Exp Ther.

[31] Chen F, Hersh B M, Conradt B, Zhou Z, Riemer D (2000). Translocation of *C.elegans* CED-4 to nuclear membranes during programmed cell death.. Science.

[32] Woo JH, Kim YH, Choi YJ, Kim DG, Lee KS (2003). Molecular mechanisms of curcumin-induced cytotoxicity: induction of apoptosis through generation of reactive oxygen species, down-regulation of Bcl-xL and IAP, the release of cytochrome *c* and inhibition of Akt.. Carcinogenesis.

[33] Garrido C, Galluzzi L, Brunet M, Puig PE, Didelot C (2006). Mechanisms of Cytochrome-c release from mitochondria.. Cell Death Differ.

[34] Wang C, Youle RJ (2009). The role of mitochondria in apoptosis.. Annu Rev Genet.

[35] Lettre G, Hengartner MO (2006). Developmental apoptosis in *C.elegans*: a complex CEDnario.. Nat Rev Mol Cell Biol.

[36] Rolland S, Conradt B (2006). The role of mitochondria in apoptosis induction in *Caenorhabditis elegans*: more than just innocent bystanders?. Cell Death Differ.

[37] Twomey C, McCarthy JV (2005). Pathways of apoptosis and importance in development.. J Cell Mol Med.

[38] Jiang X, Wang X (2004). Cytochrome-c mediated apoptosis.. Ann Rev Biochem.

[39] Kim HE, Du F, Fang M, Wang X (2005). Formation of apoptosome is initiated by Cytochrome-c induced dATP hydrolysis and subsequent nucleotide exchange on Apaf-1.. PNAS.

[40] Shaha C (2006). Apoptosis in *leishmania* species and its relevance to disease pathogenesis.. Indian J Med Res.

[41] Chen L, Rao KV, He YX, Ramaswamy K (2002). Skin stage Schistosomula of *Schistosoma mansoni* produce an apoptosis inducing factor that can cause apoptosis of T cells.. J Biol Chem.

[42] Lee N, Bertholet S, Debrabant A, Muller J, Duncan R (2002). Programmed cell death in unicellular protozoan parasite Leishmania.. Cell Death Differ.

[43] Debrabant A, Lee N, Bertholet S, Duncan R, Nakhasi HL (2003). Programmed cell death in trypanosomatids and other unicellular organisms.. Int J Parasitol.

[44] Jaso-Friedmann L, Leary JH, Evans DL (2000). Role of nonspecific cytotoxic cells in the induction of programmed cell death of pathogenic protozoans: participation of Fas ligand-Fas receptor system.. Exp Parasitol.

[45] Zmasek CM, Zhang Q, Ye Y, Godzik A (2007). Surprising complexity of the apoptosis network.. Genome Biol.

[46] USDA web site. Available: www.ba.ars.usda.gov/nematology/nem-basics.html. Accessed on 2011 Feb 1

[47] Bowman, Dwight D (2009). Georgis' Parasitology for veterinarians; Ninth Ed. Saunders, an imprint of Elsevier Inc.St. Louis, Missouri..

[48] Mes TH, Eysker M, Ploeger HW (2007). A simple, robust and semi-automated parasite egg isolation protocol.. Nat Protoc.

[49] Quignon F, De Bels F, Koken M, Feunteun J, Ameisen JC (1998). PML induces a novel caspase-independent death process.. Nat Genet.

[50] Jäättelä M (2004). Multiple cell death pathways as regulators of tumor initiation and progression.. Oncogene.

[51] Arnoult D, Parone P, Martinou JC, Antonsson B, Estaquier J (2002). Mitochondrial release of apoptosis inducing factor occurs downstream of cytochrome c release in response to several pro apoptotic stimuli.. J Cell Biol.

[52] Riccardi C, Nicoletti I (2006). Analysis of apoptosis by propidium iodide staining and flow cytometry.. Nat Protoc.

[53] Salganic RI (2001). The benefits and hazards of antioxidants: controlling apoptosis and other protective mechanisms in cancer patients and the human population.. J Am Coll Nutr.

[54] Polman K, Stelma FF, Gryseels B (1995). Epidemiological application of circulating antigen detection in a recent Schistosoma mansoni focus in northern Senegal.. Am J Trop Med Hyg.

[55] Sanderson L, Bartlett A, Whitfield PJ (2002). In vitro and in vivo studies on the bioactivity of a ginger [Zingiber officinale] extract towards adult schistosomes and their egg production.. J Helminthol.

[56] Acala-Canto Y, Velarde FI, Lopez HS, Mora JG, Alberti-Navarro A (2006). Effect of a cysteine protease inhibitor on Fasciola hepatica [liver fluke] fecundity, egg viability, parasite burden, and size in experimental infected sheep.. Parasitol Res.

[57] Meier P, Finch A, Evan G (2000). Apoptosis in development.. Nature.

