# Supplementary figures and images for: Caspase Dependent Programmed Cell Death in Developing Embryos: A Potential Target for Therapeutic Intervention against Pathogenic Nematodes
Source: PLoS Negl Trop Dis. 2011 Sep 13;5(9):e1306. doi: 10.1371/journal.pntd.0001306 (PMC3172199; doi:10.1371/journal.pntd.0001306)

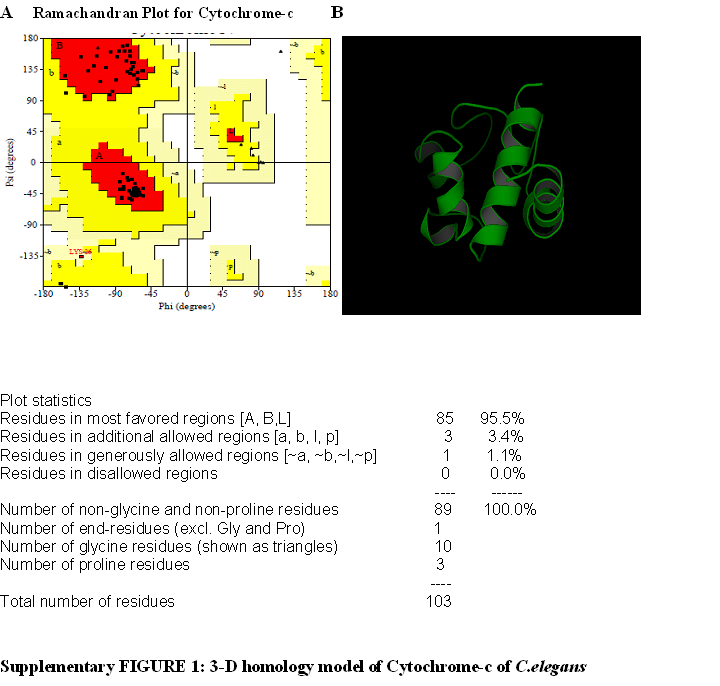

Supplement: Figure S1 — 3-D homology model of Cytochrome-c of C.elegans. (A) Ramachandran plot of the modeled structure for Cytochrome-c of C.elegans. (B) Ribbon drawing of modeled Cytochrome-c of C.elegans. The amino acid sequence of Cytochrome-c of C. elegans (target) was retrieved from the sequence database of NCBI (P19974) and its 3-D structure was generated by homology modeling, using the academic version of MODELLER9v6 software. 2B4Z was taken as a template for the modeling. (TIF) [file pntd.0001306.s001.tif]

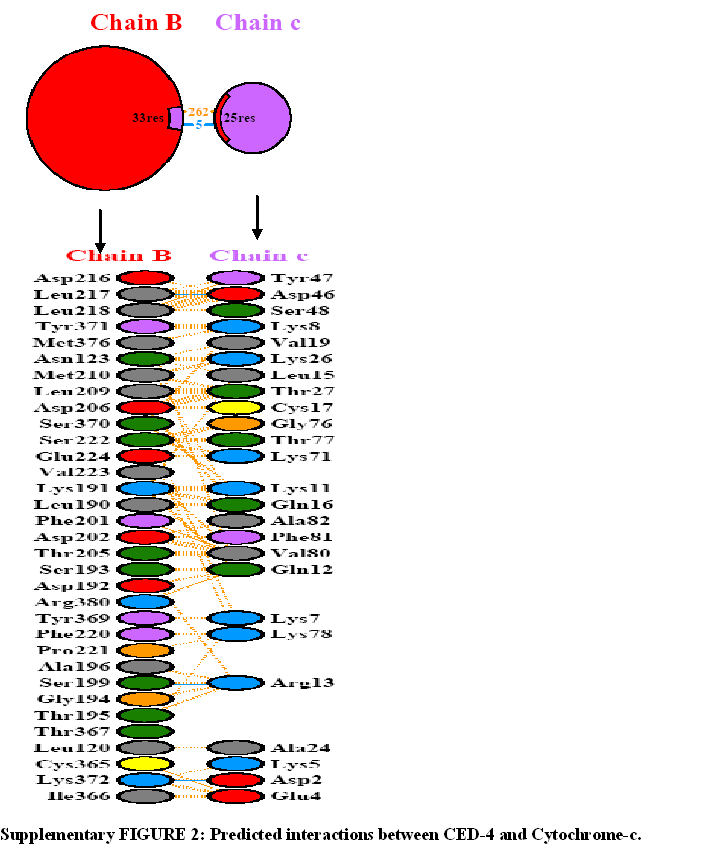

Supplement: Figure S2 — Predicted interactions between CED-4 and Cytochrome-c. The molecular docking between CED-4 and Cytochrome-c revealed 5 hydrogen bonds (Blue lines) and 262 hydrophobic interactions (Orange lines) involving 33 residues of CED-4 and 25 residues of Cytochrome-c. (TIF) [file pntd.0001306.s002.tif]

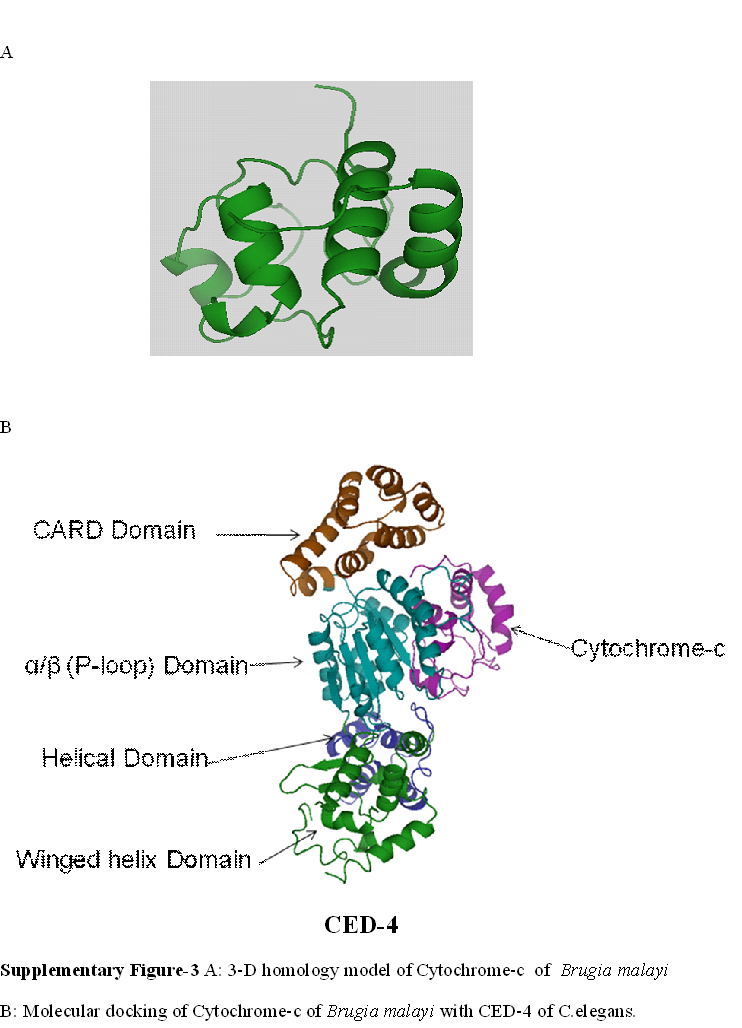

Supplement: Figure S3 — (A) Ribbon drawing of modeled Cytochrome-c of Brugia malayi . The amino acid sequence of Cytochrome-c of human filarial parasite Brugia malayi (target) was retrieved from the sequence database of NCBI (Accession NO. XP_001897096) and it's 3-D structure was generated by homology modeling, using the academic version of MODELLER9v6 software as described above. 1CCR was taken as a template for the modeling. (B) Interaction of Cytochrome-c of Brugia malayi with α/β (P-loop) ATP binding domain of CED-4 is shown. (TIF) [file pntd.0001306.s003.tif]
